# Comparison of Prime Editing and CRISPR-Cas Mediated HDR in Correcting CFTR-F508del Mutation in Patient iPSC-Derived Airway Basal Cells

**DOI:** 10.3390/ijms27198616

**Published:** 2026-09-26

**Authors:** Olga Volodina, Anna Demchenko, Ekaterina Kondrateva, Arina Anuchina, Oxana Ryzhkova, Valeriia Kovalskaia, Vyacheslav Tabakov, Elena Amelina, Alexander Lavrov, Sergey Kutsev, Svetlana Smirnikhina

**Affiliations:** 1 Laboratory of Genome Editing, Research Centre for Medical Genetics, Moscow 115522, Russia; 2 The Shared Resource Centre “Genome”, Research Centre for Medical Genetics, Moscow 115522, Russia; 3 The Shared Resource Centre “Biobank”, Research Centre for Medical Genetics, Moscow 115522, Russia; 4 Laboratory of Cystic Fibrosis, Research Institute of Pulmonology, Moscow 115682, Russia

**Keywords:** gene editing, CRISPR-Cas, prime editing, gene therapy, cystic fibrosis, F508del, *CFTR*

## Abstract

Gene editing therapy represents a promising strategy to permanently cure cystic fibrosis (CF). This paper presents the results of correcting the most common CF mutation, F508del, in the *CFTR* gene in airway basal cells (BCs) obtained from patients, using both classical CRISPR-Cas9-mediated homology-directed repair (HDR) and prime editing techniques. Our findings demonstrate that both methods achieve mutation correction with efficiencies reaching up to 18.9% of alleles in successfully transfected cells. However, the CRISPR-Cas9 approach results in insertions and deletions (indels) in approximately 15% of alleles at the editing site, whereas prime editing induced only minimal indel events. Functional analysis evaluating the effectiveness of gene editing revealed that prime editing of the F508del mutation in the *CFTR* gene—using the pegRNA4 combined with the PE2-NG variant—restores CFTR channel conductance, comparable to triple therapy with CFTR modulators (ivacaftor/tezacaftor/elexacaftor). While both prime editing and CRISPR-HDR resulted in successful correction of the CFTR-F508del mutation in patient BCs, prime editing exhibited a low frequency of unintended modifications both within and outside the editing locus, making this approach a promising candidate for the development of gene therapy strategies for CF.

## 1. Introduction

Cystic fibrosis (CF) is a rare monogenic autosomal recessive disease caused by mutations in the *CFTR* gene (in this work, mutations refer to pathogenic and likely pathogenic variants). The *CFTR* gene encodes the chloride ion channel responsible for regulating chloride transport across epithelial cell membranes [1]. The number of individuals with CF worldwide exceeds 100,000, with approximately 4000 living in Russia [2], over 33,000 registered in the USA [3], and more than 56,000 in Europe [4]. The most prevalent mutation globally is F508del (c.1521_1523delCTT, p.Phe508del), which involves a deletion of three nucleotides (CTT) and the loss of phenylalanine at position 508 of the CFTR protein. This deletion disrupts the protein’s proper folding, leading to its degradation in the endoplasmic reticulum and preventing its transport to the cell membrane [5,6,7,8]. Despite the high efficacy of current modulator therapies for CF patients with the F508del mutation (the lifespan of patients treated by CFTR modulators is expected to be in the 60s or 70s), treatment remains lifelong and expensive, often accompanied by side effects that may necessitate discontinuation [9].

Since the 1990s, researchers have been attempting to develop gene therapies for CF; however, no treatments have yet received approval for clinical use. Clinical trials have generally demonstrated limited effectiveness and short-term benefits, primarily because initial approaches were based on gene replacement strategies [10]. In addition to the limited lifespan of a functional exogenous copy of the *CFTR* gene, there has been (and continues to be) a challenge related to the low efficiency of delivering genetic constructs to the epithelial cells of the respiratory tract in patients with cystic fibrosis. Unlike these methods, gene editing offers the advantage of directly correcting mutations within the genome, providing the potential for long-lasting, possibly lifelong, therapeutic effects. Meanwhile, it should be noted that the delivery problem remains unresolved.

Genome editing encompasses a range of molecular genetic techniques that utilize programmable nucleases. Traditional methods, such as CRISPR-Cas systems, typically induce a double-strand break (DSB) at a specified location in the DNA—such as near a mutation that needs correction. Following the creation of this DSB, the cell repairs it through one of two primary pathways: non-homologous end joining (NHEJ) or homology-directed repair (HDR). When repair occurs via HDR, an external repair template is introduced to facilitate homologous recombination and accurate correction.

Recent advancements in CRISPR-Cas technology have led to modifications that do not produce DNA DSBs, which enhances both the safety and efficiency of genome editing. One such innovation is prime editing, first described in 2019 [11]. The key advantage of prime editing lies in its use of a Cas9 nickase fused with reverse transcriptase. This allows for the direct synthesis of a DNA correction template at the target site without requiring a DSB. The system employs a specially designed guide RNA called pegRNA (prime editing guide RNA). PegRNA contains the spacer that recognizes the target DNA sequence, along with two additional variable segments: the primer binding site (PBS), which serves as the initiation point for reverse transcription, and the reverse transcription (RT) template, which provides the sequence for the desired genetic correction. These components enable the localized synthesis of a repair template directly at the site of a single-stranded DNA break, facilitating precise genome editing with higher safety and efficiency.

Approximately 1500 clinical trials related to cystic fibrosis (CF) have been initiated [12], part of which focus (<5%) on gene replacement therapy. However, to date, no clinical trials involving drugs based on genome editing techniques for treating CF have been launched. Nonetheless, several research teams are conducting relevant in vitro studies. Around 50 scientific publications have reported on the preclinical development of genome-editing-based therapies for CF [13,14,15,16,17,18,19,20,21,22,23,24,25,26,27,28,29,30,31,32,33,34,35], with most targeting the correction of the most common CF mutation, F508del.

The goal of this study is to compare the effectiveness of CRISPR-HDR and prime editing in correcting the F508del mutation in CF patients. Additionally, we aim to assess the restoration of CFTR channel function following editing in patient-derived lung organoids and to evaluate potential on-target and off-target undesirable edits.

This study provides evidence that the prime editing method can effectively correct the F508del mutation in the *CFTR* gene in airway basal cells derived from iPSCs obtained from cystic fibrosis patients. Additionally, forskolin-induced swelling analysis demonstrated the restoration of CFTR channel conductance in lung organoids derived from edited basal cells. This approach will be further evaluated in comprehensive preclinical studies.

## 2. Results

### 2.1. Correction of the F508del Mutation in the CFTR Gene in CF Patient Cell Lines Using CRISPR-HDR and Prime Editing

Two genome editing strategies were employed to correct the F508del mutation in the *CFTR* gene: the CRISPR-Cas method involving double-strand breaks (DSBs)—hereafter referred to as the CRISPR-HDR—and prime editing. For the CRISPR-HDR, the nuclease eSpCas9(1.1) (hereafter spCas9(1.1)) was utilized. This engineered nuclease exhibits similar on-target activity to wild-type SpCas9 but significantly reduced off-target effects due to amino acid substitutions K848A, K1003A, and R1060A [36]. A single guide RNA (sgRNA) was designed specifically to target the F508del mutation site within the *CFTR* gene. In this study, two different single-stranded DNA (ssODN) donor molecules were used, each complementary to the break site on different DNA strands (Supplementary Table S1). For the prime editing strategy, two nickases were employed: SpCas9 with a PAM sequence of NGG (used in PEmax) and SpCas9-NG with PAM NG (used in PE2-NG). The first variant included two pegRNAs (pegRNA1 and pegRNA2), while the second incorporated five pegRNAs (pegRNA3–7) (Supplementary Table S1).

Plasmids and donor molecules were transfected into airway basal cells derived from three patients with cystic fibrosis, all of whom are homozygous for the F508del mutation in the *CFTR* gene (patients P1, P2, and P7). These cells were obtained from induced pluripotent stem cells (iPSCs) created from the patients’ fibroblasts. The scheme of the experiment is outlined in Figure 1A. In the CRISPR-HDR experiments, the transfection efficiency was 31.5%, 26.5%, and 21.5% for BC lines from patients P1, P2, and P7, respectively (*n* = 6). In prime editing experiments, the transfection efficiency was 10.4%, 5.1%, and 13.5% for BC lines from patients P1, P2, and P7, respectively (*n* = 6). After 48–72 h, DNA was extracted from the cells, and the *CFTR* gene fragment was amplified. Deep targeted sequencing was then performed, and the results were analyzed using the CRISPResso2 software [37] or its modified version. Using CRISPR-HDR, the average coverage depth was 159,172 reads (from 41,228 to 324,287 reads). With prime editing, the average coverage depth was 135,337 reads (from 28,066 to 309,544 reads).

During the assessment of editing efficiency, a considerable diversity of alleles was observed within the samples (Supplementary Figure S1). These included the unmodified (wild-type) sequence of the *CFTR* locus, as well as alleles containing the desired CTT insertion that corrects the F508del mutation. The results showed both perfect editing, where only the CTT insertion was present, and imperfect editing, where the CTT insertion was accompanied by additional changes such as deletions or nucleotide substitutions within the same allele. Additionally, some alleles lacked the target insertion but exhibited other undesirable modifications.

The results of correcting the F508del mutation in the *CFTR* gene in BCs from three cystic fibrosis patients using CRISPR-HDR are shown in Figure 1B (the figure displays data that have been normalized for transfection efficiency; the unnormalized data can be found in Supplementary Table S2). The CRISPR-HDR variant with the donor molecule ssODN2 achieved an average mutation correction rate of 20% in the cells of patient P1 (*p* < 0.01 compared to the untransfected control) and 13.9% in the cells of patient P2 (*p* < 0.01 compared to the untransfected control). In patient P7’s cells, this editing approach showed results indistinguishable from the untransfected control, with an average correction efficiency of approximately 1.2%. The CRISPR-HDR variant with the donor molecule ssODN1 did not produce a statistically significant increase in the proportion of alleles with the corrected mutation across cells from all three donors. Additionally, there was a statistically significant difference in mutation correction efficiency between the variants using ssODN1 and ssODN2 in the cells of patients P1 and P2.

The proportion of perfect editing of the F508del mutation in the BCs of patients P1 and P2 for the ssODN2 variant was notably high—77.7% and 86.2%, respectively (Figure 1B). In other words, we observed that 13.8–22.3% of the alleles, in addition to the F508del correction, contained undesired changes (such as indels or substitutions) that would disrupt the allele. This indicates a high degree of accuracy in the HDR when using ssODN2.

Pooled data on the efficiency and accuracy of correcting the F508del mutation in the *CFTR* gene using CRISPR-HDR with the ssODN2 donor molecule across BC lines from three patients are summarized in Figure 1C (Supplementary Figure S2). The average correction efficiency was 13.8% for overall editing, with 11.2% representing precise edits free of unintended nucleotide changes.

The results of prime editing are presented in Figure 1D. As shown in Figure 1D, there are notable differences in mutation correction efficiency among cells from different patients. Analyzing the spectrum of undesirable changes associated with mutation correction (the spectrum of imperfect editing), it was found that most such alterations are caused by single nucleotide substitutions within the editing locus. These single nucleotide substitutions are most likely due to errors that occur during amplification and sequencing processes.

The prime editing variants pegRNA1 + PEmax, pegRNA5 + PE2-NG, and pegRNA6 + PE2-NG showed a significant increase in the proportion of alleles with corrected mutations across the BC lines of all three patients. However, the pegRNA1 + PEmax variant in cells from patient P2 exhibited lower accuracy, with only 36% of the alleles showing perfect correction. The pegRNA5 + PE2-NG variant was more effective than pegRNA6 + PE2-NG in cells from two of the three patients, whereas pegRNA6 + PE2-NG demonstrated superior efficiency only in cells from one patient. Although the pegRNA4 + PE2-NG variant was significantly effective in cells from two patients, overall, it exhibited relatively high efficiency and reproducibility across samples. Based on these findings, it can be concluded that the prime editing variants pegRNA4 + PE2-NG and pegRNA5 + PE2-NG demonstrated the highest reproducibility across different patient cells and maintained relatively high editing efficiency. When averaging data from all three patient cell lines, the corrected F508del mutation in the *CFTR* gene—normalized for transfection efficiency (which ranged from 3% to 15%)—showed an average correction rate of 18.9% with 93% accuracy using pegRNA4 + PE2-NG. In comparison, pegRNA5 + PE2-NG achieved an average correction efficiency of 17.4% with 94% accuracy (Figure 1E).

Based on the experimental results obtained from BC lines of three CF patients homozygous for the F508del mutation in the *CFTR* gene, the average correction efficiency using CRISPR-HDR and the donor molecule ssODN2 was approximately 13.8%. In comparison, the prime editing variants, pegRNA4 + PE2-NG and pegRNA5 + PE2-NG, achieved correction efficiencies of 18.9% and 17.4%, respectively (Figure 1F). Although prime editing demonstrated a higher correction efficiency, there were no statistically significant differences compared to the editing efficiency achieved with CRISPR-HDR. Meanwhile, CRISPR-HDR editing also introduces additional unwanted changes at the target locus alongside the F508del correction, which could potentially render the modulator-responsive allele (carrying F508del) insensitive to therapies by causing indels.

### 2.2. Restoration of CFTR Channel Conductance Following CFTR F508del Mutation Editing in Lung Organoids Derived from CF Patients

Lung organoids (LOs) obtained from CF patients can be used to evaluate CFTR channel conductance. The organoid FIS test not only predicts patient responses to therapy [38] but also serves to confirm the restoration of channel conductance after *CFTR* mutation editing. To assess the extent of CFTR channel function recovery, lung organoids were derived from patients’ BCs following editing of the F508del mutation in the *CFTR* gene.

Since the prime editing method is associated with fewer editing errors (Figure 1E,F), it was decided to evaluate the CFTR channel conductance restoration only after prime editing. The evaluation of the restoration of the CFTR channel function in the LO after prime editing in the groups where the sequencing results showed the highest editing efficiency was performed using the FIS method. For this purpose, the cells were first thawed after editing, and then LOs were formed from them. Freezing of the edited cells was essential for technical processing and sequencing of samples. At this stage, it was not possible to obtain organoids from all edited cells (conditions). As a result, the channel function could be measured only in some conditions because, due to the requirement for a relatively long cell culture period to achieve the necessary cell count, not all edited cells (conditions) successfully formed LOs (Supplementary Table S3). Untransfected (non-edited) cells from the same patients were used as a negative control. Untransfected cells treated with triple combination therapy with the CFTR modulator elexacaftor/tezacaftor/ivacaftor (ETI therapy) was used as a positive control. It is important to note that Supplementary Table S3 presents data on the efficiency of correction of the F508del mutation without normalization for transfection efficiency. Since it was not possible to select transfected and/or edited cells (the plasmids with editors did not contain a reporter), channel conductance analysis was performed in organoids obtained from a mixture of edited and unedited cells.

Based on the results of the FIS analysis, only one of the examined sample groups (P7-2-4.3, after editing with the pegRNA4 + PE2-NG variant) exhibited a statistically significant (**—*p* < 0.0001) increase in the LO (luminal opening) area in response to forskolin, with a 21% increase relative to the negative control group. In the remaining groups, there was no statistically significant response to forskolin following editing (Figure 2). Representative images of LOs from the FIS assay are shown in Supplementary Figure S3A,B.

The data obtained may suggest the existence of a threshold number of CFTR-expressing cells, above which the organoid begins to respond to forskolin. However, further experiments are needed to confirm this hypothesis—for example, by mixing cells from a healthy donor with those from a patient who has a homozygous mutation F508del in varying proportions, and then conducting FIS analysis.

Another possible explanation is that the proportion of CFTR-expressing cells within the organoids may actually be less than 10%. Consequently, it was not possible to select transfected cells or enrich the population of transfected cells in this study. To test this hypothesis, cells frozen after editing were thawed and cultured, and DNA was extracted. A fragment of the *CFTR* gene was amplified, followed by deep targeted sequencing. The resulting data were analyzed using the CRISPResso2 program. Three samples were re-analyzed (Supplementary Table S4). As shown in the table, the proportion of edited alleles in the repeated analysis decreased significantly.

In this study, unedited BCs from patients P1 and P7, which received additional triple therapy, were used as ETI controls (Figure 2). On average, in the ETI control, the normalized area of organoids from patient P7 increased by 22%, compared to the size of the LOs before the addition of forskolin and ETI, and that of P1 by 46%. In the sample post-editing with the pegRNA4 + PE2-NG variant (P7-2-4.3), an increase of 21% compared to the size of the LOs before the addition of forskolin was observed. In other analyzed samples, no statistically significant increase in organoid area was observed after editing and forskolin treatment compared to the negative control. The difference between P7 ETI control and edited P7 cells was not statistically significant (*p* > 0.9999). Thus, the restoration of CFTR channel conductivity after editing achieved levels comparable to those observed with triple therapy.

It was also important in this study to compare the conductivity of the CFTR channel after editing with that of a healthy donor. As shown in Figure 2, on average, organoids from healthy donors increase in size by twofold when stimulated with forskolin, whereas organoids derived from edited cells increase by only 1.2 times. Available data suggest that prime editing restores CFTR channel conductance, but not to normal levels. At this stage, it is challenging to precisely determine the extent to which channel conductance is restored to normal.

In summary, we can conclude that editing the F508del mutation in the *CFTR* gene using the pegRNA4 + PE2-NG variant restores CFTR channel function comparable to the effect achieved with triple CF modulators therapy. Thus, this study demonstrates the high efficiency of prime editing in addressing the F508del mutation in the *CFTR* gene, enabling at least a 20% restoration of CFTR channel conductivity.

### 2.3. Unintended Editing of Genome Editing Methods at the F508del Mutation Locus in the CFTR Gene

To estimate the frequency of unintended modifications at the editing site, deep targeted sequencing data were analyzed following the editing process. Undesirable modifications were defined as indels and nucleotide substitutions within a 10-nucleotide window upstream and downstream of the expected DNA nuclease cleavage site. In this section, the results are presented without adjusting for transfection efficiency. The frequency of nucleotide substitutions and indels in the samples was normalized to the corresponding frequency of these events in the negative (untreated) samples. As noted earlier, undesirable DNA alterations at the editing locus can occur on the same allele that undergoes the desired mutation correction. This section reports the frequencies and spectrum of these undesirable DNA changes at the editing locus without considering successful mutation correction.

Analysis of the frequency of undesirable changes in the *CFTR* gene editing locus using CRISPR-HDR in BC lines revealed an increase following editing in cells derived from patients P1 (both editing variants) and P2 (editing with ssODN2). In all BC lines studied, the frequency of indels increased after editing—except for cells from patient P7 edited with ssODN2—while the frequency of nucleotide substitutions remained roughly comparable to untransfected control samples. A significant rise in the proportion of undesirable changes was observed, primarily due to an increase in indels in BCs from patient P1 with both donor molecules (*p* < 0.01) and in BCs from patient P2 following editing with ssODN2 (*p* < 0.01) (Figure 3A). The ratio of the total number of wanted edits to the total number of unwanted edits is presented in Supplementary Table S2.

The results showing an increase in indels at the editing locus generally correlate with the frequency of F508del mutation correction (Figure 1B). The study demonstrates that, in the BCs of patient P1, the corrected mutation frequency—adjusted for transfection efficiency—reaches 20% when using ssODN2 (*p* < 0.01). In patient P2’s BCs, this value is 13.9% (*p* < 0.01).

Focusing solely on indels, the frequency in P1’s BCs with ssODN2 is 5.6%, while in P2’s BCs it is 4.4%. These figures do not account for transfection efficiency. In experiments, the average transfection efficiencies were 32% for P1 and 26% for P2. When normalized for transfection efficiency, the indel frequency in P1’s BCs with ssODN2 is approximately 18%, and in P2’s BCs, about 17%. Correspondingly, the mutation correction rates are 20% for P1 and 13.9% for P2, with the error-free correction rates being even lower—15.5% and 11.8%, respectively. Thus, the frequency of undesirable modifications (indels plus alleles with imperfect editing) exceeds that of error-free mutation correction: for P1, the combined undesirable modifications amount to 22.5% (18% indels plus 4.5% imperfect corrections) compared to 15.5% for error-free corrections; for P2, it is 19.1% versus 11.8%.

Taken together, the results of editing the F508del mutation in the *CFTR* gene using CRISPR-HDR, as demonstrated in BCs from cystic fibrosis patients, indicate that in the cells of two out of three patients, there is a significant increase in both mutation editing frequency and the introduction of undesirable changes at the editing locus. Moreover, the proportion of alleles with undesirable modifications exceeds the proportion of alleles with successfully corrected mutations. These results were not replicated in the BCLs of the third patient.

To estimate the frequency of undesirable changes at the editing locus following prime editing, deep targeted sequencing data were also analyzed post-experiment. Similarly to the CRISPR-HDR approach, indels as well as nucleotide substitutions within a 10-nucleotide region upstream and downstream of the intended DNA nicking site were quantified. This analysis was performed without accounting for transfection efficiency. The results presented here include the frequencies and spectrum of undesirable DNA modifications at the editing locus, excluding mutation correction.

When analyzing the frequency of undesirable alterations at the *CFTR* gene editing locus after prime editing in BC lines, it is noteworthy that these are primarily due to single-nucleotide substitutions, with only 0.2% attributable to indels. Furthermore, the proportion of undesirable changes in unedited samples did not differ significantly from those in edited samples, both in terms of nucleotide substitutions and indel frequencies (Figure 3B). Therefore, it can be concluded that prime editing in this study did not increase the proportion of undesirable modifications at the editing locus.

When comparing the efficiency of correcting the F508del mutation in the *CFTR* gene using CRISPR-HDR and prime editing, the results indicate that both approaches are similarly effective. CRISPR-HDR with ssODN2 achieved a correction rate of 13.8% across alleles, while prime editing with pegRNA4 + PE2-NG and pegRNA5 + PE2-NG variants achieved correction rates of 18.9% and 17.4%, respectively. This suggests that both methods could be potentially viable therapies. However, significant differences were observed in the frequency of undesirable changes at the editing locus between these two approaches (Figure 3C). Specifically, editing with CRISPR-HDR and ssODN2 resulted in an accumulation of indels, likely due to incorrect DSB repair via the NHEJ mechanism, whereas prime editing did not show an increase in indel frequency relative to control values.

If we assume that nucleotide substitutions result from amplification and/or sequencing errors rather than editing, then these substitutions can be disregarded in further analysis. In this context, the proportion of changes introduced into DNA during CRISPR-HDR editing is 4%, corresponding to indels. The average transfection efficiency for BC lines from three patients in CRISPR-HDR editing experiments was 26.5%. Consequently, it can be estimated that CRISPR-HDR induces indels in approximately 15% of alleles, accounting for transfection efficiency. This rate exceeds the mutation correction efficiency of 13.8% and the error-free mutation correction efficiency of 11.2% observed with the same method. Based on these findings, the tested CRISPR-HDR approach with ssODN for therapeutic purposes appears unfeasible.

In contrast, the proportion of indels after prime editing averaged 0.2%, which did not differ from the untransfected control.

In conclusion, the analysis indicates that prime editing of the F508del mutation in the *CFTR* gene does not generate unwanted changes at the editing site. Unlike the CRISPR-HDR method that introduces DSBs, prime editing demonstrates a higher potential for safety and efficiency, making it a more promising approach for gene therapy.

### 2.4. Assessment of Off-Target Activity of Genome Editing Methods Following F508del Mutation Correction in the CFTR Gene

To evaluate the non-specific effects of CRISPR-HDR genome editing, we conducted a search and analysis of predicted off-target sites in BC lines both prior to and following editing.

Using the sgCFTR spacer sequence, we identified 783 potential off-target sites. All these candidate sites were annotated and subjected to detailed analysis. Since all possible genomic changes induced by editing are somatic and do not result in hereditary diseases or inheritance, our focus was on variants that could potentially contribute to oncogenesis. Consequently, we restricted our analysis to off-target sites located within genes associated with malignant neoplasms and performed thorough investigations of these loci.

In this study, ten potential off-target sites within genes linked to malignant tumors were identified for the CRISPR-HDR sgRNA. Due to the complex sequences of the gene fragments involved, it was possible to design amplification primers for only five genes: *BTLA*, *IL1RAP*, *WNK2*, *ARID4A*, and *TOX2*. Therefore, assessment of undesired modifications was confined to these genes. DNA was isolated from samples collected both before and after editing. The regions predicted as potential off-target sites were amplified using primers listed in Supplementary Table S5. Subsequently, deep sequencing of these amplicons was performed to detect any unintended modifications. On average, the coverage of off-target loci for the CRISPR-HDR sgRNA spacer was 52,534 reads (from 11,356 to 147,960).

Figure 4A summarizes the detected undesirable changes at potential off-target sites, indicating the frequencies of indels and nucleotide substitutions. The proportion of undesirable alterations in the analyzed genes did not differ between the BC samples before and after editing. Most of these changes were single-nucleotide substitutions, while the proportion of indels did not exceed 0.2% of all reads from deep targeted sequencing. Furthermore, there was no significant difference in the frequency of these changes between edited and unedited BC samples. In other words, no increase in the levels of nucleotide changes or indels above the background was observed. Analysis of the spectrum of undesirable alterations in the specified genes revealed that all detected indels and nucleotide substitutions also appeared in the negative controls. Therefore, all identified undesirable changes at the predicted off-target sites are most likely not the result of incorrect editing. Instead, they are probably due to amplification and/or sequencing errors or are a consequence of random mutagenesis.

As with CRISPR-HDR, the non-specific activity of prime editing in the genome following correction of the F508del mutation in the *CFTR* gene was evaluated by analyzing the frequency of changes at predicted off-target loci. It is known that prime editing does not require DNA DSBs, which can lead to off-target effects; according to the existing literature, the non-specific effects of prime editing are close to zero [11,34,39].

Since the spacer sequence of the CRISPR-HDR sgRNA was identical to the spacer sequences of pegRNA1 and pegRNA2, the search for potential off-target sites for these pegRNAs was not repeated. Instead, previously obtained results were used. For the spacers of pegRNA3 and pegRNA4, 2618 potential off-target sites were identified. For the spacers of pegRNA5, pegRNA6, and pegRNA7, 1510 potential off-target sites were detected. All identified potential off-target sites were annotated and analyzed. Variants in genes not associated with cancer were excluded from the analysis.

In this study, the most effective prime editing variants were PE2-NG with pegRNA4 and pegRNA5. Therefore, assessment of non-specific changes was performed only in samples edited with these variants.

A total of 14 potential off-target sites were identified for pegRNA4. Due to the complex sequence of the analyzed gene fragments, primers could only be designed for amplification of the genes *AGBL4*, *CBX1*, *DLEU2*, *HEATR6*, *HSP90B1*, *NOD1*, and *PARD3*; primers for other loci could not be selected. Consequently, assessment of undesirable modifications was limited to these specific genes. DNA samples were collected both before and after editing, and fragments corresponding to the predicted off-target sites were amplified using the primers listed in Supplementary Table S5. Subsequently, deep sequencing of these amplicons was performed. On average, the coverage of off-target loci for pegRNA4 were 166,022 reads (from 73,664 to 346,213), for pegRNA5—98,022 (from 33,370 to 171,107).

Figure 4B summarizes the data on undesirable alterations detected at the potential off-target sites, reporting the frequencies of indels and nucleotide substitutions. The proportion of undesirable changes in these genes remained consistent in the BC samples before and after editing. The majority of observed modifications were single-nucleotide substitutions, while indels did not exceed 0.35% of the total reads from deep targeted sequencing. No significant differences were observed between edited and unedited BC samples.

Analysis of the spectrum of undesirable changes revealed that all identified modifications—both indels and nucleotide substitutions—also appeared in negative control samples. Therefore, the detected changes at the predicted off-target sites are most likely not a consequence of off-target editing but are attributable to sequencing or amplification errors or are the result of random mutagenesis.

At the next stage of the study, off-target sites for pegRNA5 were analyzed using the same algorithm applied to pegRNA4. The summarized results are shown in Figure 4C. The proportion of undesirable mutations in the analyzed genes did not differ in BC samples before and after editing, with the majority being single nucleotide substitutions. Additionally, the frequency of indels did not exceed 0.15% of all reads from deep targeted sequencing and showed no significant difference between edited and unedited BC samples. No differences were observed in the frequency or spectrum of undesirable changes between the edited samples.

In summary, it can be concluded that no clear off-target editing was found at the selected sites using pegRNA4 + PE2-NG and pegRNA5 + PE2-NG variants. The frequencies and types of undesirable modifications in the edited cells are comparable to those observed in unedited control samples.

## 3. Discussion

This study represents the initial phase in the development of gene-therapy-based treatments for cystic fibrosis (CF), which will subsequently undergo preclinical and clinical testing. The development of these therapies has the potential to cure CF or substantially improve patients’ quality of life, thereby increasing their life expectancy. Additionally, the cost of these treatments is expected to be significantly lower than lifelong therapy with CFTR modulators, which could reduce healthcare expenses for providing medication to tens of thousands of CF patients. Preliminary estimates suggest that therapies targeting the F508del mutation in the *CFTR* gene could be suitable for over 50% of CF patients in Russia and more than 70% worldwide.

When developing therapeutic approaches, a key consideration is determining what level of efficiency is sufficient to advance to the next stage of development. After analyzing the available published data, this study adopted a threshold of 10% of alleles. In other words, if the mutation correction efficiency reaches 10% or higher, it is considered likely to produce meaningful clinical improvements in patients. The 10% threshold was selected based on several factors. Studies have demonstrated that not all cells in a population need to express a functional CFTR channel to achieve normal chloride transport; only a proportion of cells is sufficient. For example, research by Farmen SL et al. [40] showed that just 20% of CFTR-expressing cells are enough to normalize chloride ion transport. Similarly, Johnson LG et al. [41] found that even 6–10% of CFTR-expressing cells can restore chloride transport to normal levels. Additionally, clinical studies with ivacaftor reveal that a therapeutic response can occur with less than 10% of normal CFTR function restored [42]. Therefore, correcting the mutation in at least 10% of cells should theoretically restore CFTR expression across the cell population and normalize chloride transport. In the current work, the exact proportion of cells in which the F508del mutation has been corrected through genome editing is unknown. However, it is understood that in cells containing two mutated alleles, genome editing often affects only one allele, leaving the second intact—particularly when repair occurs via the HDR mechanism [43,44]. Accordingly, it can be assumed that a 10% correction rate at the allele level corresponds approximately to 10% of cells carrying at least one corrected allele. However, there is still a possibility that both alleles are edited in a single cell, which would result in a lower overall proportion of edited cells. We do not yet understand the exact correlation between the proportion of CFTR-expressing cells and the clinical outcome, which underscores the need for functional tests in vivo.

In this study, the correction efficiency of the F508del mutation in the *CFTR* gene using CRISPR-HDR and prime editing was comparable (Figure 1F). Previously, we conducted extensive experiments to identify the most effective CRISPR-HDR variants [45] and prime editing approaches [46]. Only the most efficient variants, tested further for both effectiveness and safety, were included in this study. Our comprehensive work demonstrates that the most effective CRISPR-HDR and prime editing variants show comparable efficiencies in correcting F508del (Figure 1F). However, we acknowledge that not all possible genome editing options have been tested, so we cannot definitively conclude that these approaches have equal efficiencies.

Figure 1 illustrates data on the correction efficiency of the F508del mutation, normalized for transfection efficiency. Supplementary Table S2 provides additional data without normalization. It is important to note that the editing efficiency, when not normalized, is below 5% in all cases, which falls short of the 10% threshold necessary for effective in vivo treatment. We are optimistic that selecting the optimal delivery system for in vivo editing will enhance overall efficiency. In our in vitro experiments, the average transfection efficiency of the prime editor was only 9.7%. Our ongoing research is focused on developing a highly efficient editor delivery method; we are currently testing both viral (adenovirus, lentivirus, virus-like particles) and non-viral (vesicles and lipid nanoparticles) delivery systems to identify the most effective and safe option for in vivo applications.

We utilized electroporation to deliver CRISPR-HDR plasmids and lipofection (Lipofectamine LTX) for prime editing, resulting in different transfection efficiencies (26.5% for CRISPR-HDR vs. 9.7% for prime editing). We refrained from using electroporation for prime editing due to significant variability in transfection rates across cell lines from different patients, leading to low reproducibility. While Lipofectamine is less efficient, it provides greater stability. The low transfection efficiency resulted in suboptimal editing rates for prime editing when not normalized for transfection efficiency. Our primary objective at this stage is to identify an optimal in vivo delivery method that will ensure overall editing efficiency exceeds 10%.

The main reason for the lack of reliable differences in mutation correction efficiency between the two methods under investigation is the considerable variability in the obtained results (Figure 1F). Specifically, the efficiency when using CRISPR-HDR ranged from 0.23% to 35.6%, while with prime editing (pegRNA4 + PE2-NG) it varied from 0.1% to 90%, and with pegRNA5 + PE2-NG from 0.1% to 86% (after normalization for transfection efficiency) (Figure 1D). Despite including a relatively large number of both technical and biological replicates and standardizing all experimental stages, we were unable to minimize these pronounced differences in editing efficiency.

If we compare the results obtained in this work with those published previously, we can state that this is the third study focused on correcting the F508del mutation in the *CFTR* gene using prime editing. Geurts MH et al. [19] demonstrated a very low efficiency of mutation correction in intestinal organoids derived from CF patients—only single organoids with corrected mutations were obtained. The efficiency of prime editing was lower than that of the traditional CRISPR-HDR method. Meanwhile, a study published in 2025 showed that the correction efficiency of F508del in primary human airway epithelial cells from three patients homozygous for the *CFTR* F508del mutation was 25%. However, a high frequency of indels—6.5%—was observed at the editing locus [47]. Overall, the mutation correction efficiency achieved in this work (Figure 1F) is comparable, with the best result reaching 18.9%. When comparing the pegRNA sequences in our work to those in Sousa’s study [47], it is evident that we used shorter variable regions (PBS and RT). The editor used in the referenced study included an additional gene, *hMLH1dn* (encoding a mismatch repair inhibitor), on a separate plasmid, which enhances editing efficiency [48]. However, this approach has two notable disadvantages: (1) increased size of the editor, complicating delivery into cells; and (2) because MLH1dn is expressed from a separate cassette, its activity is not limited to the editing site but affects the entire genome. This interference can inhibit crucial DNA repair mechanisms and may potentially lead to cell death or oncogenic processes [49]. However, there is currently no direct evidence linking transient MLH1dn expression with oncogenicity. In contrast, the editor employed in this work does not contain additional factors that interfere with DNA repair.

When analyzing the literature data, it can be concluded that this is the first study of its kind in which the F508del mutation was specifically edited in BCs, followed by the formation of lung organoids and a functional assessment of CFTR channel conductivity using the FIS method (Figure 2). In previous studies in this area, either different editing targets were used—for example, organoids were edited directly, and the FIS assay was performed after editing [19,50]—or the editing was conducted in 2D cultures, with conductivity assessed by measuring ion currents in Ussing chambers [24,47,51]. Therefore, direct comparison between the results of this study and previously published data is not entirely appropriate. However, most of the cited studies included comparisons with positive controls: either with samples from healthy donors [19,24,47,51] or through the addition of CFTR modulators [51].

Overall, the level of CFTR channel conductance restoration following editing (Figure 2) is comparable to results reported in other studies on F508del mutation correction. Suzuki S. et al. [51] observed a restoration of CFTR channel conductance by Ussing chamber to approximately 40% of normal after editing with ZFNs, with a correction efficiency of 10.6% ± 2.6% of alleles. Vaidyanathan S. et al. [24] reported a correction efficiency of 28% ± 5% of alleles, resulting in channel conductance assessed by Ussing chamber restored to between 20% and 50% of normal. Similarly, Sousa A.A. et al. [47] demonstrated that editing the F508del mutation increased CFTR channel conductance to about 50% of normal (by Ussing chamber), with a correction efficiency of 25% of alleles.

It is important to highlight that, in this study, a significant restoration of CFTR channel conductivity was achieved despite only 10.27% of alleles being edited (Figure 2, Supplementary Table S3). The low level of editing primarily resulted from limited transfection efficiency of the editor into BCs. Improving the delivery of genetic constructs is expected to enhance editing efficiency, leading to more substantial mutation correction and greater restoration of CFTR channel conductance.

The notable reduction in the proportion of edited cells can largely be attributed to cell death following transfection (Supplementary Table S4). The initial assessment of editing efficiency was conducted 72 h after transfection, whereas subsequent evaluations—such as FIS and NGS—were performed much later. This delay involved growing the cells to the desired density, cryopreserving them, thawing, expanding, forming organoids, and only then conducting the analyses.

It is important to highlight the study by Sousa A.A. et al. [47], which reported the highest success rates in correcting the F508del mutation in the *CFTR* gene using prime editing. The authors demonstrated that with a mutation correction efficiency of approximately 25% across alleles, indels were observed in 6.5% of alleles. This relatively high rate of undesirable modifications at the editing site is primarily attributable to the editor they used—PE6c. This editor is a modified version of PE3 [11], which induces an additional single-stranded DNA break on the opposite strand, effectively creating a double-stranded break with sticky ends through the use of an extra guide RNA. The addition of this guide RNA generally enhances editing efficiency, as supported by the existing literature [11]. However, a significant drawback of this approach is the increased frequency of indels—undesirable insertions and deletions that disrupt the reading frame and can inactivate the gene [11]. In the referenced study [47], the occurrence of such indels was 6.5%. In contrast, the editor used in the present work results in only 3.9% of additional changes, which are predominantly benign single-nucleotide substitutions. The average frequency of indels with our editor is approximately 0.2% (Figure 3B,C and Figure 4B,C). Therefore, although Sousa A.A. et al. achieved slightly higher correction efficiencies, the editor employed in this study offers superior safety profiles while maintaining comparable efficacy.

As noted earlier, because prime editing does not induce DSBs, the proportion of indels arising after editing is extremely low [11], which is confirmed by the present study (Figure 3B,C and Figure 4B,C). Because the average efficiency of prime editing delivery was relatively low—around 10% of cells—we can infer that the actual levels of off-target changes at both the editing locus and across the genome could be higher than those reported here. We are actively testing approximately a dozen viral and non-viral delivery systems aimed at significantly improving delivery efficiency. A limitation of this study is that off-target effects were assessed solely based on predicted sites and those with minimal mismatches to the sgRNA or pegRNA spacer sequences. We recognize that off-target effects may also occur at loci with little or no homology to the spacer. A more thorough investigation of off-target modifications, including those at less homologous sites, is planned for future work.

Some studies have reported that prime editing can occasionally introduce undesired modifications into off-target loci. For example, Sousa et al. [47] found that four out of 96 analyzed genes showed a 1.5-fold or greater increase in indel frequency in edited cells compared to unedited controls when correcting the F508del mutation in the *CFTR* gene in primary airway epithelial cells from cystic fibrosis patients. Importantly, the indel frequency at each of these off-target sites in edited cells did not exceed 0.1%.

Furthermore, the use of unbiased methods—which do not consider the homology between the guide RNA spacer and the genomic locus—enables the identification of additional off-target sites. For example, TIPE-seq can detect up to 1% off-target sites (including indels and substitutions) in the genome of cells edited with PE2 and PEmax nucleases [52]. It has also been shown that using double CRISPR-Cas9 nickases can lead to large deletions or inversions within a 10 kb region surrounding the target sites [53]. Additionally, there is a continuously expanding database of off-target sites validated in experimental cell systems. Although this database currently contains limited data on base and prime editing, existing evidence suggests that both methods can induce off-target modifications [54]. While Cas9-based nickases generally show a significantly lower rate of off-target effects—often below 1%—these off-target edits still occur and should be carefully considered, especially for therapeutic applications. Therefore, unbiased off-target detection methods followed by thorough validation in cell-based experiments are essential for ensuring safety and efficacy.

Before conducting clinical trials, it is essential to determine the most effective delivery method at the preclinical stage. The vector must effectively penetrate the target cells (airway basal cells) in vivo, and the entire prime editor must be encapsulated within it. In addition to selecting the type of vector, it is crucial to establish the route of administration. Since cystic fibrosis is a multisystem disease, systemic administration of the drug would be ideal; however, there are concerns about whether it will adequately reach the airway basal cells. Given that lung damage is the predominant issue, it is also important to consider alternative routes of administration, such as endotracheal (intratracheal or inhalation) delivery. These matters should be addressed within the framework of preclinical in vivo studies.

Furthermore, since cystic fibrosis is associated with irreversible organ damage, such as fibrotic changes in the lungs, it is vital to initiate treatment immediately after diagnosis. Many countries conduct neonatal screenings for cystic fibrosis, which allows for treatment to commence at a pre-symptomatic stage, thereby improving the prognosis for affected patients.

## 4. Materials and Methods

### 4.1. Obtaining of Airway Basal Cells from iPSCs

Basal cells were differentiated from induced pluripotent stem cells (iPSCs), all three cell lines confirmed to be homozygous for the F508del mutation (this mutation was verified in parental fibroblasts, iPSCs, and basal cells). The iPSCs were generated from dermal fibroblasts using non-integrating vectors—either the CytoTune™ iPS 2.0 Sendai Reprogramming Kit (Thermo Fisher Scientific, Waltham, MA, USA) or the ReproRNA™ OKSGM Kit (STEMCELL Technologies, Vancouver, BC, Canada). The resulting iPSCs were free of transgenes. Characteristics of the patient lines P1 and P7 are described in previous publications [55,56]; the characterization of line P2 is available at: https://hpscreg.eu/cell-line/RCMGi013-A (accessed on 23 September 2026).

Airway basal cells (BCs) were obtained from human iPSCs as described previously [57]. In brief, iPSCs were differentiated into definitive endoderm through treatment with Activin A, then patterned into anterior foregut endoderm. Next, anterior foregut endoderm cells were differentiated into NKX2.1+ lung progenitor cells. A population of airway basal cells was subsequently isolated from these progenitors by cell sorting for the basal cell surface marker CD271 (NGFR). Finally, the sorted BCs were expanded in culture on pre-coated with Matrigel (Corning, Corning, NY, USA) in PneumaCult™-Ex Plus Medium (STEMCELL Technologies, Canada), 1 μM A83-01 (Tocris, Bristol, UK), 1 μM DMH1 (Tocris, UK), 1 μM hydrocortisone (Sigma Aldrich, St. Louis, MO, USA), 50 U/mL penicillin, and 50 μg/mL streptomycin (Paneco, Moscow, Russia). BCs were passaged every 4–5 days using Versene solution (Paneco, Russia).

### 4.2. Maintenance of BC Lines

BCs were maintained on culture plastic pre-coated with Matrigel (Corning, USA) in a basal cell medium (PneumaCult™-Ex Plus Medium (StemCell Technologies, Canada), 1 μM A83-01 (Tocris, UK), 1 μM DMH1 (Tocris, UK), 1 μM hydrocortisone (Sigma Aldrich, USA)). BCs were passaged every 4–5 days using Versen solution (Paneco, Russia).

### 4.3. Plasmids for Correction of F508del Mutation in the CFTR Gene

Benchling software (https://www.benchling.com/crispr) (accessed on 23 September 2026) was used to design the sgRNA spacer for CRISPR-HDR. The sgRNA spacer sgCFTR (5’-ACCATTAAAGAAAATATCAT-3’) was selected directly for the locus with the F508del mutation in the *CFTR* gene. In this case, the PAM sequence for sgCFTR appears only in the presence of the F508del mutation, i.e., the Cas9 nuclease can produce DSB only in the presence of the mutation. The spacer sequence was synthesized as two overlapping oligonucleotides and cloned into the plasmid vector eSpCas9(1.1) (was a gift from Feng Zhang (Addgene plasmid #71814; http://n2t.net/addgene:71814 (accessed on 23 September 2026); RRID:Addgene_71814)). Thus, the plasmid spCas9(1.1)-sgCFTR was obtained.

The design of DNA molecules encoding pegRNAs was carried out using the open source software PE-Designer [58], as well as the pegLIT tool (https://peglit.liugroup.us) (accessed on 23 September 2026). The pegRNA design was described in detail in our recent work [46]. Briefly, pegRNAs for nickases with PAM sequences NGG and NG were selected with short RTTs (10–19 nt) and PBSs (12–14 nt), considering the efficiency data from the publication of Anzalone et al. [11] and taking into account the GC content of the sequences. The sequences of the variable fragments of pegRNA are presented in Supplementary Table S1. To assemble plasmids containing pegRNA, we used the plasmid pU6-pegRNA-GG-acceptor (was a gift from David Liu (Addgene plasmid #132777; http://n2t.net/addgene:132777 (accessed on 23 September 2026); RRID:Addgene_132777)). In this way, the plasmids pU6-pegRNA1, pU6-pegRNA2, pU6-pegRNA3, pU6-pegRNA4, pU6-pegRNA5, pU6-pegRNA6, pU6-pegRNA7 were obtained.

### 4.4. Design of Single-Stranded Oligodeoxyribonucleotides (ssODNs)

When selecting ssODNs for the repair of DSB DNA resulting from the nuclease activity of Cas9, we were guided by general recommendations regarding the total length of the molecule and homology arms [59], as well as the site of the expected DNA cleavage by the Cas9 enzyme. Two ssODNs were selected: ssODN1 and ssODN2, homologous to different strands of the cleaved DNA. The sequences of the selected ssODNs are given in Supplementary Table S1.

### 4.5. Transfection of BC Lines

Transfection of BCs homozygous for F508del in *CFTR* gene with genetic constructs for CRISPR-HDR was performed by electroporation using the Neon Transfection System (Thermo Fisher Scientific, USA). BCs were removed from the culture plastic with Versene solution, the cells were counted on a Countess II FL automatic cell counter (Thermo Fisher Scientific, USA) and centrifuged at 150× *g* for 5 min. The pellet was resuspended in electroporation buffer at a concentration of 0.5 × 10^6^ cells per 100 μL. 1.2 μg of spCas9(1.1)-sgCFTR plasmid and 18 pM ssODN were added to the BC suspension containing 90 × 10^3^ cells. Electroporation was performed in 3 steps using 10 μL cuvette tips (Neon Transfection System 10 μL) with the following settings: voltage 1290 mV, amplitude 5 s, 2 pulses. Then the cells were placed on culture plastic in the BC medium. To control the transfection efficiency, BC were also electroporated with pEGFP-C1 plasmid (Clontech, Mountain View, CA, USA). 72 h after transfection, the cells were harvested. Cells electroporated with the pEGFP-C1 plasmid were analyzed using a flow cytometer. DNA was isolated from the remaining cells, and the target locus was analyzed using deep targeted amplicon sequencing.

Transfection of BCs homozygous for F508del in *CFTR* gene for prime editing was performed by lipofection using the commercial Lipofectamine LTX kit (Thermo Fisher Scientific, USA). The day before lipofection, BCs were passaged onto a 48-well plate at 10 × 10^3^ cells per well in basal cell medium. Then, lipofection was performed with plasmid combinations (287 ng of plasmid with the editor (pCMV-PEmax was a gift from David Liu (Addgene plasmid #174820; http://n2t.net/addgene:174820 (accessed on 23 September 2026); RRID:Addgene_174820); pCMV-PE2-NG was a gift from Yongsub Kim (Addgene plasmid #159977; http://n2t.net/addgene:159977 (accessed on 23 September 2026); RRID:Addgene_159977) and 63 ng of plasmid with pegRNA), with the addition of 1.25 μL Lipofectamine LTX. The experiment was carried out on three BC lines with homozygous (F508del/F508del) mutation in the *CFTR* gene. Transfection efficiency was assessed on a CytoFLEX flow cytometer 72 h after lipofection. In addition, 72 h after lipofection, the cells were removed from the culture plastic, DNA was isolated from half of the cells using the standard phenol-chloroform method, and the other half of the cells were further cultured.

### 4.6. Amplification of CFTR Gene Fragments and Their Deep Targeted Sequencing

To analyze the efficiency of changing the target locus of the *CFTR* gene as a result of genome editing in BC lines, 48–72 h after transfection, the cells were removed and DNA was isolated, which was used for PCR amplification of the *CFTR* gene locus in the region of the F508del mutation. Primer sequences and amplicon lengths are given in Supplementary Table S5. The stages and conditions of PCR were as follows: 1 cycle of initial denaturation (3 min, 95 °C); 35 cycles including denaturation (30 s, 95 °C), primer annealing (30 s, 60 °C) and elongation (30 s, 72 °C); and 1 cycle of resynthesis of the amplicon end regions (5 min, 72 °C). Deep targeted sequencing of amplicons was performed at The Shared Resource Centre “Genome” of the Research Centre for Medical Genetics using the MiSeq System (Illumina, San Diego, CA, USA). Libraries were prepared using KAPA HiFi HotStart ReadyMix and KAPA Hyper Prep PCR kits, KAPA dual-index adapters, 15 μM KAPA UDI Adapter Plate (96) (all Roche Sequencing Solutions, Cape Town, South Africa) were used, sequencing was performed on a MiSeq instrument (Illumina, USA) using a MiSeq v3 600. Sequencing data were analyzed using the CRISPResso2 [37] or its modification created at the Research Centre for Medical Genetics (https://github.com/GlushkovNikita/CRISPResso2UI (accessed on 23 September 2026)).

The pipeline performs quality control, alignment, and precise quantification of indels and substitutions compared to the provided reference sequence. For CRISPResso2 analysis of CRISPR-HDR experiments, the following parameters were applied: Edition tool—Cas9; minimum homology for alignment to an amplicon—60%; quantification window size—6 bp; and the option “ignore short reads.” Reads with events occurring only once were excluded from the analysis, while the remaining reads were included. Normalization to the negative control was not performed. For CRISPResso2 analysis of prime editing experiments, the same parameters were applied. Spacer sequence of pegRNA was used as sgRNA sequence.

### 4.7. Evaluation of the Unintended Editing of Genome Editors

The unintended editing of CRISPR-HDR and prime editing at the editing locus was assessed based on the analysis of deep targeted sequencing data for the *CFTR* gene fragment, which was previously used to determine the editing efficiency.

The search and analysis of potential off-target sites for the sgRNA spacer for CRISPR-HDR (ACCATTAAAGAAAATATCAT) and spacers used in pegRNAs (ACCATTAAAGAAAATATCAT, CCATTAAAGAAAATATCATT, TTCATCATAGGAAACACCAA) was performed using the Cas-OFFinder program (http://www.rgenome.net/cas-offinder/) (accessed on 23 September 2026) [60]. The following parameters were used for the search: Mismatch Number: 2 or less, DNA Bulge Size: 2 or less, RNA Bulge Size: 2 or less. DNA was isolated from the samples before and after editing, and fragments predicted as potential off-target sites were amplified using the primers listed in Supplementary Table S5. Then, deep targeted sequencing of the resulting amplicons was performed as described above. The analysis was performed using the CRISPResso2 program.

### 4.8. Obtaining Lung Organoids from BCs

Transfected or control BCs were removed from the culture plastic using Versen solution (Paneco, Russia), the cells were counted on a Countess II automatic cell counter and centrifuged at 150× *g* for 5 min. The pellet was resuspended in undiluted Matrigel (Corning, USA) that had been cooled to 4 °C at a concentration of 400 cells per 1 μL and transferred dropwise with a volume of 20 μL into the wells of a 48-well plate. The drops were allowed to solidify for 40 min in an incubator, after which the medium for culturing lung organoids was added. The medium for lung organoids consisted of 75% IMDM (Thermo Fisher Scientific, Waltham, MA, USA), 25% Ham’s F12 (Paneco, Moscow, Russia), 100× B-27 (Thermo Fisher Scientific, Waltham, MA, USA), 200× N2 (Paneco, Moscow, Russia), 0.05% bovine serum albumin solution (Sigma Aldrich, St. Louis, MO, USA), 0.45 mM 1-thioglycerol (Sigma Aldrich, St. Louis, MO, USA), 100× GlutaMAX (Thermo Fisher Scientific, Waltham, MA, USA), 0.05 mg/mL L-ascorbic acid (Sigma Aldrich, St. Louis, MO, USA) and 100× penicillin–streptomycin (Paneco, Moscow, Russia), 10 ng/mL FGF7 (R&D Systems, Minnesota, MN, USA), 10 ng/mL FGF10, 10 ng/mL EGF (R&D Systems, Minnesota, MN, USA) and 3 µM CHIR99021. The formed organoids were observed on days 5–7.

### 4.9. Forskolin-Induced Swelling of Lung Organoids

Edited BCs were thawed and used to generate lung organoids. Freezing of the edited cells was necessary and was due to the waiting time for deep targeted sequencing data, based on the results of which samples for analysis had to be selected. Forskolin-induced swelling (FIS) of lung organoids (LO) was carried out on the 7th day after the formation of LOs from BCs. The day before the analysis, the organoids were passaged into a 96-well plate with a drop volume of 3 μL in the medium for lung organoids. On the day of analysis, phase-contrast images were acquired using an automated Lionheart FX imager (BioTek, Winooski, VT, USA). Subsequently, forskolin (Sigma-Aldrich, USA) was added to the wells containing LOs at a final concentration of 10 μM, incubated for 24 h and an image was obtained on an automated Lionheart FX imager. The analysis of organoid size over time was performed using the open-source program OrganoID, which performs segmentation and tracking of organoids through machine learning models based on a U-Net architecture [61]. The GraphPad Prism program was used to normalize the organoid areas, where the average organoid area in the group at 0 h after forskolin addition was taken as 100%.

### 4.10. Statistical Analysis

To assess the statistically significant frequency of indels and substitutions and to correct for the F508del mutation, nonparametric Mann–Whitney or Kruskal–Wallis tests (GraphPad Prism 8.0.1) were used; Bonferroni correction was taken into account for multiple comparisons. A *p*-value below the threshold (0.05) indicates significant differences between the control and test sample groups.

## Figures and Tables

**Figure 1:**
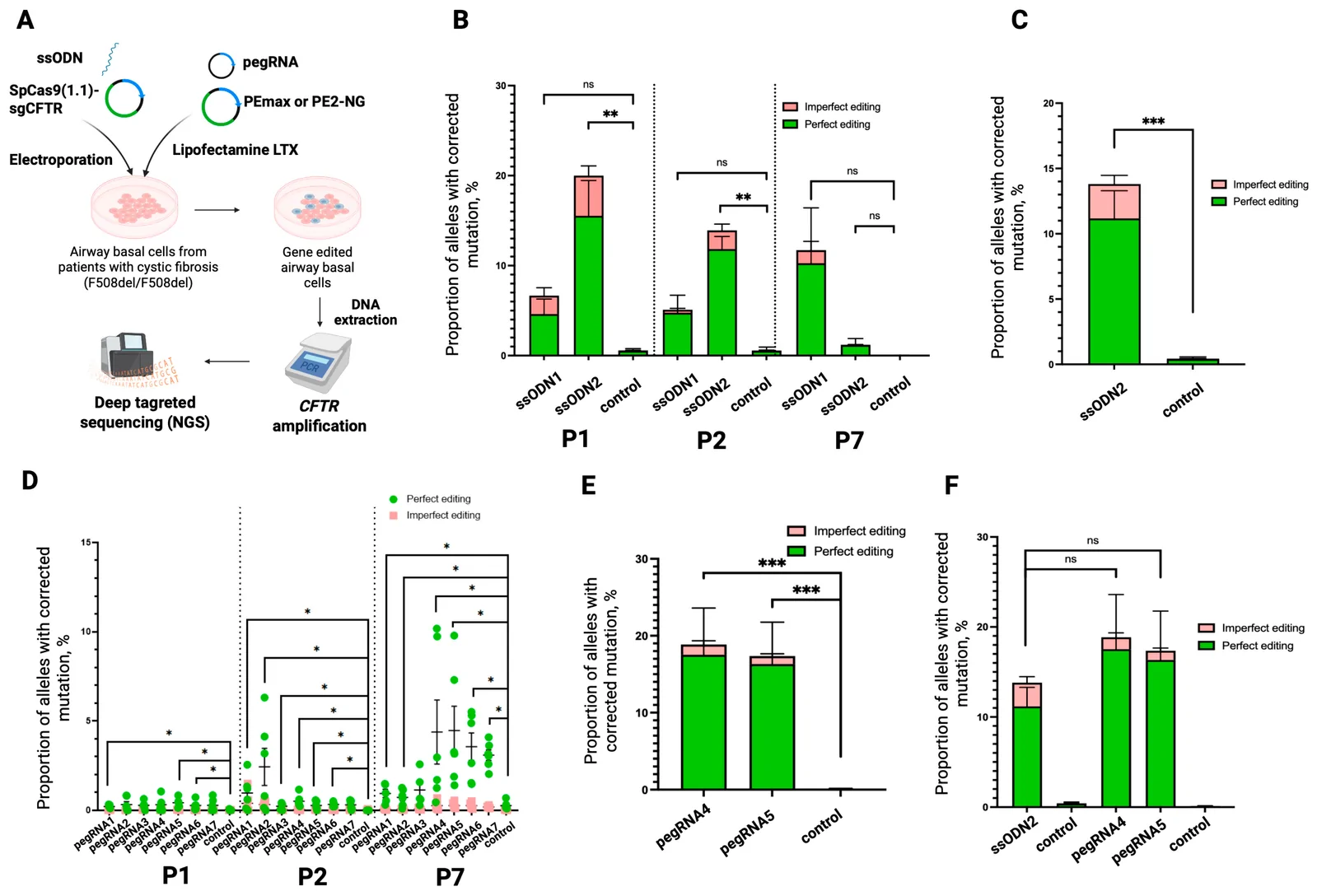
Correction of the F508del mutation in the *CFTR* gene in cystic fibrosis (CF) cell lines derived from CF patients using CRISPR-HDR and prime editing. (**A**)—Scheme of the experimental procedure on CF patient-derived cell lines. (**B**)—Proportion of alleles carrying the corrected F508del mutation in *CFTR* gene in CF patient cell lines treated with CRISPR-HDR. P1, P2, and P7 represent individual patients. Data are shown as mean ± SEM, with *n* = 3–6. (**C**)—Proportion of alleles with the corrected F508del mutation in pooled CF cell lines from multiple patients treated with CRISPR-HDR using the donor molecule ssODN2. Data are presented as mean ± SEM, with *n* = 12–15. (**D**)—Proportion of alleles with corrected F508del mutation in BC lines from CF patients following prime editing. pegRNA1–2 were used with PEmax, and pegRNA3-7 were used with PE2-NG. P1, P2, and P7 denote individual patients. Data are displayed as individual means (green circles for perfect correction, pink squares for imperfect editing), with horizontal lines indicating mean values for each correction type. Values are provided without normalization for transfection efficiency. Error bars represent SEM; *n* = 5–6. (**E**)—Proportion of alleles with corrected F508del mutation in pooled BC lines from three CF patients (data from experiments in BC lines of patients P1, P2 and P7 were merged) using prime editing with pegRNA4 + PE2-NG and pegRNA5 + PE2-NG variants. Values are provided with normalization for transfection efficiency. Data are shown as mean ± SEM, with *n* = 17–18. (**F**)—Comparison of correction efficiency in BC lines from CF patients using CRISPR-HDR with ssODN2 donor molecule (the column is signed as ssODN2 with own control) and prime editing with pegRNA4 + PE2-NG and pegRNA5 + PE2-NG variants (the columns are signed as pegRNA4 and pegRNA5 with own control). Values are provided with normalization for transfection efficiency. Data are represented as mean ± SEM, with *n* = 12–18. * *p* < 0.007, ** *p* < 0.01, *** *p* < 0.0001; ns—not significant.

**Figure 2:**
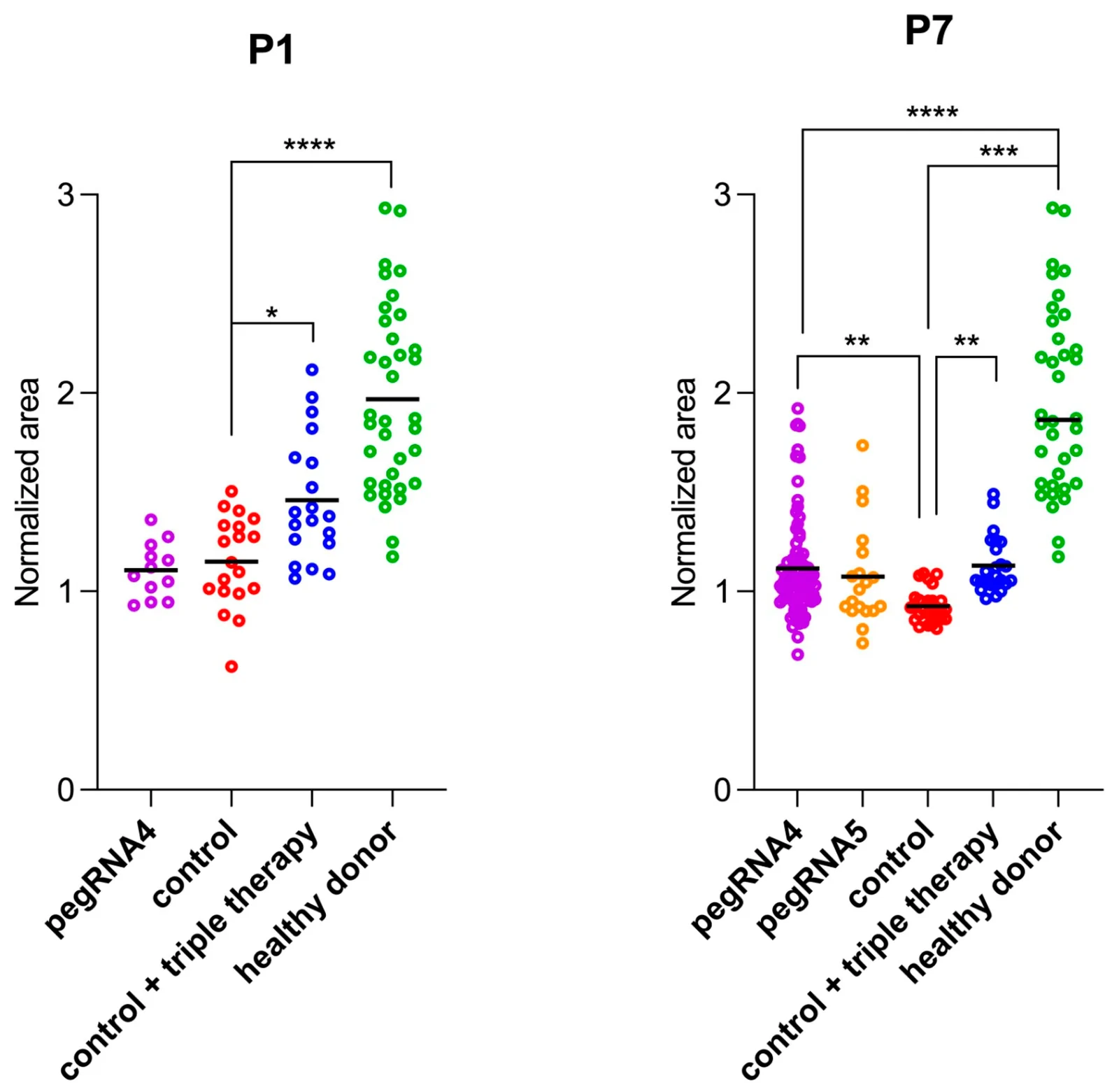
Forskolin-induced swelling of lung organoids derived from edited BCs of patients P1 and P7. The figure shows the quantification of normalized swelling areas (at time zero) of lung organoids (LOs) derived from BCs of two patients (P1 and P7) with homozygous F508del mutations in the *CFTR* gene at both 0 (before adding forskolin) and 24 h (after incubation with forskolin for 24 h). The triple therapy group consists of untransfected cells treated with a combination of CFTR modulators (ivacaftor/tezacaftor/elexacaftor, referred to as ‘control + triple therapy’). Data are presented as scatter plots with the median indicated; *n* = 7 technical replicates. Statistical significance is indicated as follows: **** *p* < 0.0001, *** *p* < 0.001, ** *p* < 0.01, * *p* < 0.05.

**Figure 3:**
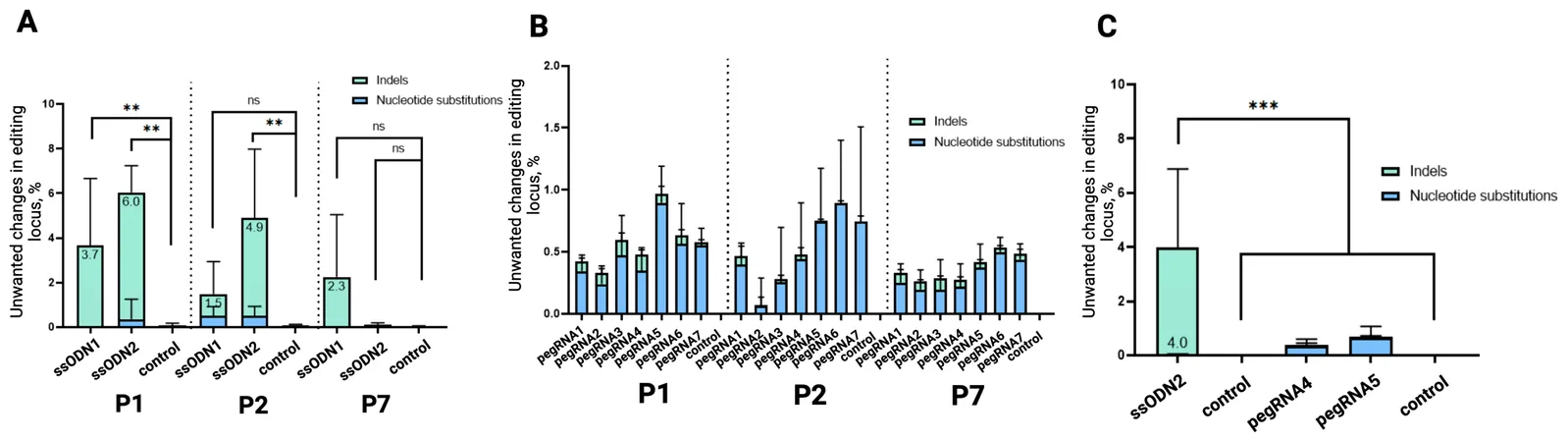
Undesirable alterations at the editing site during correction of the F508del mutation in the *CFTR* gene in BCs from CF patients. (**A**)—Percentage of alleles with undesirable changes at the *CFTR* gene editing locus following CRISPR-HDR editing in BC lines from CF patients. P1, P2, and P7 represent individual patients. Data are expressed as mean ± SEM, with a sample size of 3–6. (**B**)—Percentage of alleles with undesirable changes at the *CFTR* gene editing locus following prime editing in BC lines from CF patients. P1, P2, and P7 represent individual patients. Data are shown as mean ± SEM, with *n* = 6. (**C**)—Percentage of alleles with undesirable changes at the *CFTR* gene editing locus in pooled CF patient cell lines, comparing CRISPR-HDR editing using the ssODN2 donor molecule with prime editing employing pegRNA4 + PE2-NG and pegRNA5 + PE2-NG variants. Data are presented as mean ± SEM, with a total of 12–18 samples. ** *p* < 0.01, *** *p* < 0.0001; ns—not significant. Mann–Whitney test with Bonferroni correction was used for data in Panels (**A**,**C**), and Kruskal–Wallis test for data in Panel (**B**).

**Figure 4:**
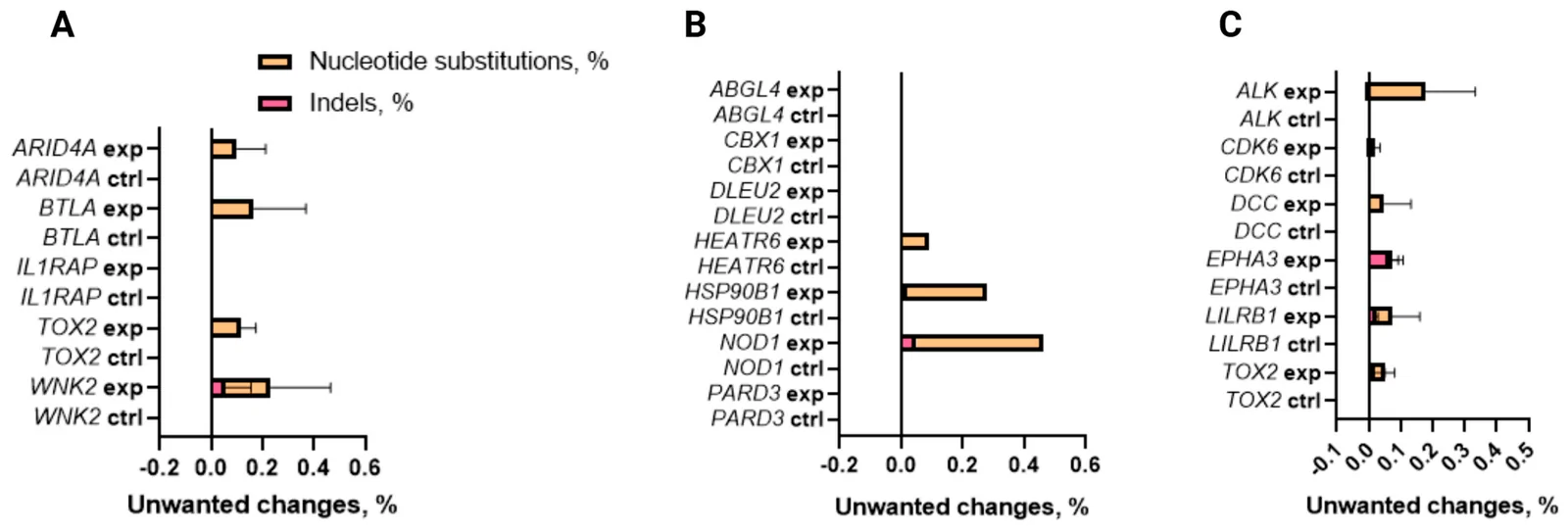
Frequencies and spectrum of undesirable DNA sequence changes at predicted off-target loci before and after editing the F508del mutation in the *CFTR* gene in CF patients’ BCs. (**A**)—For the sgCFTR spacer (CRISPR-HDR), (**B**)—for the pegRNA4 spacer, (**C**)—for the pegRNA5 spacer.

## Data Availability

Data are available on request.

## Supplementary Materials

The following supporting information can be downloaded at: https://www.mdpi.com/article/10.3390/ijms27198616/s1.

## Abbreviations

The following abbreviations are used in this manuscript:

BC	Basal cell
CF	Cystic fibrosis
DSB	Double-strand break
ETI	Elexacaftor/tezacaftor/ivacaftor
FIS	Forskolin-induced swelling
HDR	Homology-directed repair
iPSCs	Induced pluripotent stem cells
LO	Lung organoid
NHEJ	Non-homologous end joining
PBS	Primer binding site
pegRNA	Prime editing guide RNA
RT	Reverse transcription
sgRNA	Single guide RNA
ssODN	Single-stranded DNA
