# Supplementary material for: Comparison of Prime Editing and CRISPR-Cas Mediated HDR in Correcting CFTR-F508del Mutation in Patient iPSC-Derived Airway Basal Cells
Source: Int J Mol Sci. 2026 Sep 26;27(19):8616. doi: 10.3390/ijms27198616 (PMC13665346; doi:10.3390/ijms27198616)
Supplement: Supplementary file 1 [file ijms-27-08616-s001.zip › Supplementary Figures.pdf]

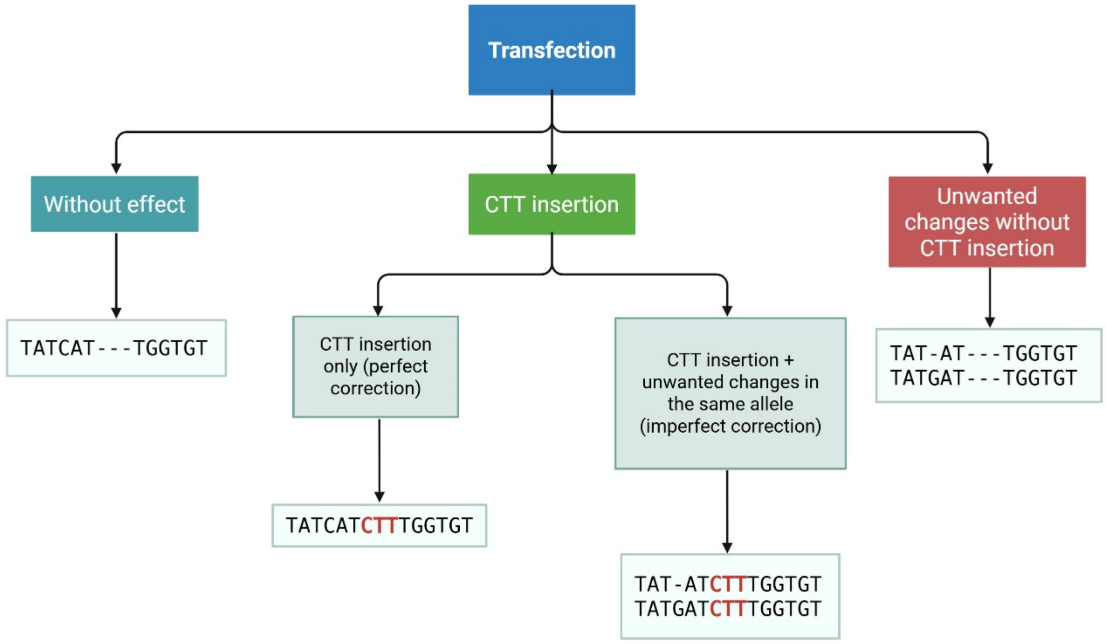

Supplementary Figure S1. Changes in the CFTR allele sequences following F508del editing, as assessed in this study. The "Unwanted changes…" shown in the figure are "examples" for illustration purposes, but not the actual unwanted sequences that were revealed by sequencing.

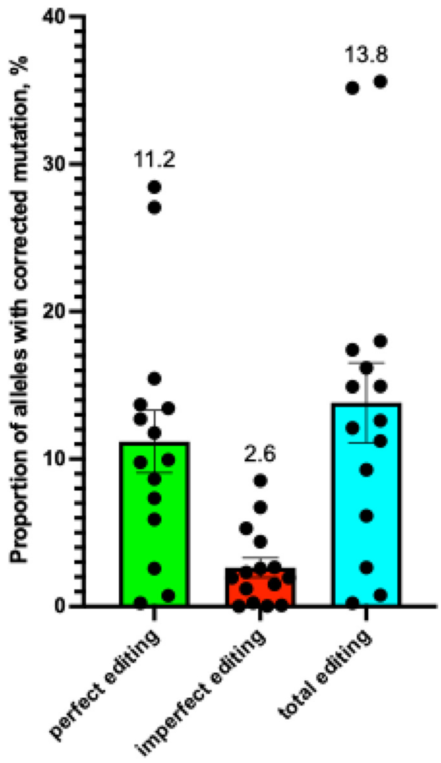

Supplementary Figure S2. Proportion of alleles with the corrected F508del mutation in pooled CF cell lines from multiple patients treated with CRISPR-HDR using the donor molecule ssODN2. Data are presented as mean ± SEM.

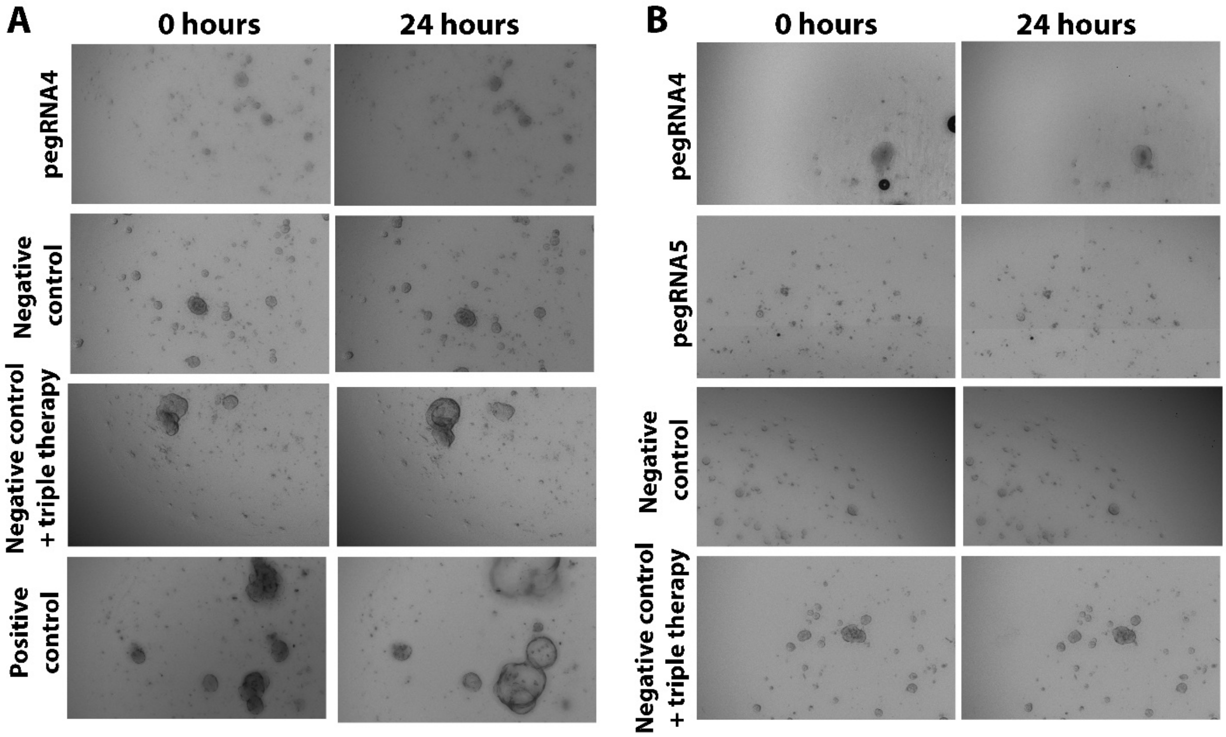

Supplementary Figure S3. Phase-contrast images of lung organoids derived from basal cells at 0 and 24 hours of the FIS assay. Magnification ×4. (A) LOs from the P1 BC line (pegRNA4 editing; negative control – untransfected cells; untransfected cells + ETI therapy; positive control – healthy donor) after prime editing, culturing, and subsequent freeze-thaw cycling, and LOs from a healthy donor BCs as a positive control. (B) LOs from the P7 BC line after prime editing, culturing, and subsequent freeze-thaw cycling
